# Toll-like receptor 4 modulation influences human neural stem cell proliferation and differentiation

**DOI:** 10.1038/s41419-017-0139-8

**Published:** 2018-02-15

**Authors:** Chiara Grasselli, Daniela Ferrari, Cristina Zalfa, Matias Soncini, Gianluigi Mazzoccoli, Fabio A. Facchini, Laura Marongiu, Francesca Granucci, Massimiliano Copetti, Angelo Luigi Vescovi, Francesco Peri, Lidia De Filippis

**Affiliations:** 1 Department of Biotechnology and Biosciences, University of Milano-Bicocca, Milan, MI Italy; 2 Department of Medical Sciences, IRCCS Casa Sollievo della Sofferenza, San Giovanni Rotondo, FG Italy; 3 Unit of Biostatistics, IRCCS Casa Sollievo della Sofferenza, San Giovanni Rotondo, FG Italy; 4 Department of Regenerative Medicine, IRCCS Casa Sollievo della Sofferenza, San Giovanni Rotondo, FG Italy

## Abstract

Toll-like receptor 4 (TLR4) activation is pivotal to innate immunity and has been shown to regulate proliferation and differentiation of human neural stem cells (hNSCs) *in vivo*. Here we study the role of TLR4 in regulating hNSC derived from the human telencephalic-diencephalic area of the fetal brain and cultured *in vitro* as neurospheres in compliance with *Good Manifacture Procedures* (GMP) guidelines. Similar batches have been used in recent clinical trials in ALS patients. We found that TLR2 and 4 are expressed in hNSCs as well as CD14 and MD-2 co-receptors, and TLR4 expression is downregulated upon differentiation. Activation of TLR4 signaling by lipopolysaccharide (LPS) has a positive effect on proliferation and/or survival while the inverse is observed with TLR4 inhibition by a synthetic antagonist. TLR4 activation promotes neuronal and oligodendrocyte differentiation and/or survival while TLR4 inhibition leads to increased apoptosis. Consistently, endogenous expression of TLR4 is retained by hNSC surviving after transplantation in ALS rats or immunocompromised mice, thus irrespectively of the neuroinflammatory environment. The characterization of downstream signaling of TLR4 in hNSCs has suggested some activation of the inflammasome pathway. This study suggests TLR4 signaling as essential for hNSC self-renewal and as a novel target for the study of neurogenetic mechanisms.

## Introduction

Stem cells in brain niches are responsible for neurogenesis and integration of new neurons into functional circuits, with inflammatory and immune system mediators playing critical roles in neurogenesis and in several diseases of the nervous system^1,2^. A family of innate immune receptors, the Toll-like receptors (TLRs), respond to the presence of minute amounts of circulating pathogen-associated molecular patterns (PAMPs) and tissue damage-related ligands (danger or damage-associated molecular patterns, DAMPs) thus activating signaling pathways that influence neural development^3,4^. These receptors are expressed in the central nervous system by microglia, perivascular dendritic cells, and neural progenitor cells (NPCs) and can respond to endogenous DAMPs^5^. In particular, TLR2 and TLR4 are present  in adult NSC and NPCs, where they exert different and contrasting functions in NPC proliferation and differentiation^6,7^. TLR2 activation, in infectious, ischemic, and inflammatory diseases, could negatively impinge on brain development by inhibition of NPC proliferation^8^. On the contrary, TLR4 activation has been shown to correlate with increased proliferation of NSC/NPC after hippocampal ischemic injury^7^. Previous studies in murine cells or animal models have shown a multifaceted role played by TLR4 in neurogenesis^9^, but the lack of a human system to study the CNS and the paucity of data on human patients have represented a roadblock to the appropriate knowledge of some pathophysiological mechanisms and to plan possible therapeutic strategies. On the other hand, there is increasing pharmacological interest in TLRs targeting in CNS pathologies^10^.

The synthetic glycolipid FP7 is a potent, selective and non-toxic TLR4 antagonist^11^, while bacterial lipopolysaccharide (LPS) is the natural TLR4 agonist. FP7 inhibits LPS–TLR4 signaling and also blocks TLR4 signal activated by endogenous DAMPs, such as high-mobility group box 1 (HMGB1) protein, with a IC_50_ in the low micromolar range, while being inactive on other TLRs, included TLR2^12^. The activation of TLR4 pathway by bacterial LPS requires the participation of the co-receptor cluster of differentiation 14 (CD14, GPI-linked or soluble) and of the adapter myeloid differentiation factor 2 (MD-2) that associates non-covalently with TLR4 to form the activated complex (LPS/MD-2/TLR4)_2_ on the cell membrane ^13^.

The TLR4 antagonism by FP7 is based on binding to MD-2 and CD14, and subsequent inhibition of LPS binding and subsequent formation of the final activated complex^11^.

Given the limited knowledge of TLR4-mediated effects on human CNS, the aim of our study was to investigate the role of TLR4 on human NSC (hNSC) irrespectively of the presence of non-neural cells. We have addressed this issue by analyzing in vitro the effects of long-term TLR4 stimulation by LPS and inhibition by the synthetic antagonist FP7 on the proliferation dynamics and cell fate of human NSCs. In particular, we used hNSCs derived from the human telencephalic-diencephalic area of the fetal brain and cultured *in vitro* as neurospheres in compliance with *Good Manufacturing Practice* (GMP) guidelines. This aspect is particularly important if considering that the use of different sources and methods to derive hNSC caused misleading interpretations in many studies^6,7^. We have established a Cell Factory and Biobank at the Hospital Santa Maria in Terni authorized from the Italian Medicines Agency (AIFA, aM 146/2016) that is currently producing fetal brain hNSCs lines characterized by a consolidated paradigm to assess their *bona fide* stemness^14^. These hNSCs have been successfully used in the recently concluded phase I trial for amyotrophic lateral sclerosis (ALS) patients^15^. In a translational perspective, the investigation of the role of TLR4 in the regulation of hNSC lines promoted as a tool for cell-mediated therapy is of utmost importance. Here we show that TLR4 is endogenously expressed in hNSC *in vitro*, is essential for their self-renewal and influences neuronal/oligodendroglial differentiation and survival in a time-dependent fashion. To note, hNSCs still retain TLR4 expression *in vivo*, after transplantation into ALS rats and immunocompromised mice, suggesting TLR4 as a novel target for future therapeutic approaches.

## Results

### TLR4 expression in human NSC

TLR2 and TLR4 receptors are both involved in the development of the inflammatory response to neuronal injury and in the capacity of modulating self-renewal and differentiation of mouse hippocampal NSCs^6–16^. However, no data are available on the expression of TLR2 and TLR4 in hNSCs. We observed by immunofluorescence that both TLR2 and TLR4 are expressed by undifferentiated hNSC and undergo downregulation with differentiation (Fig. S1).

**Fig. 1:**
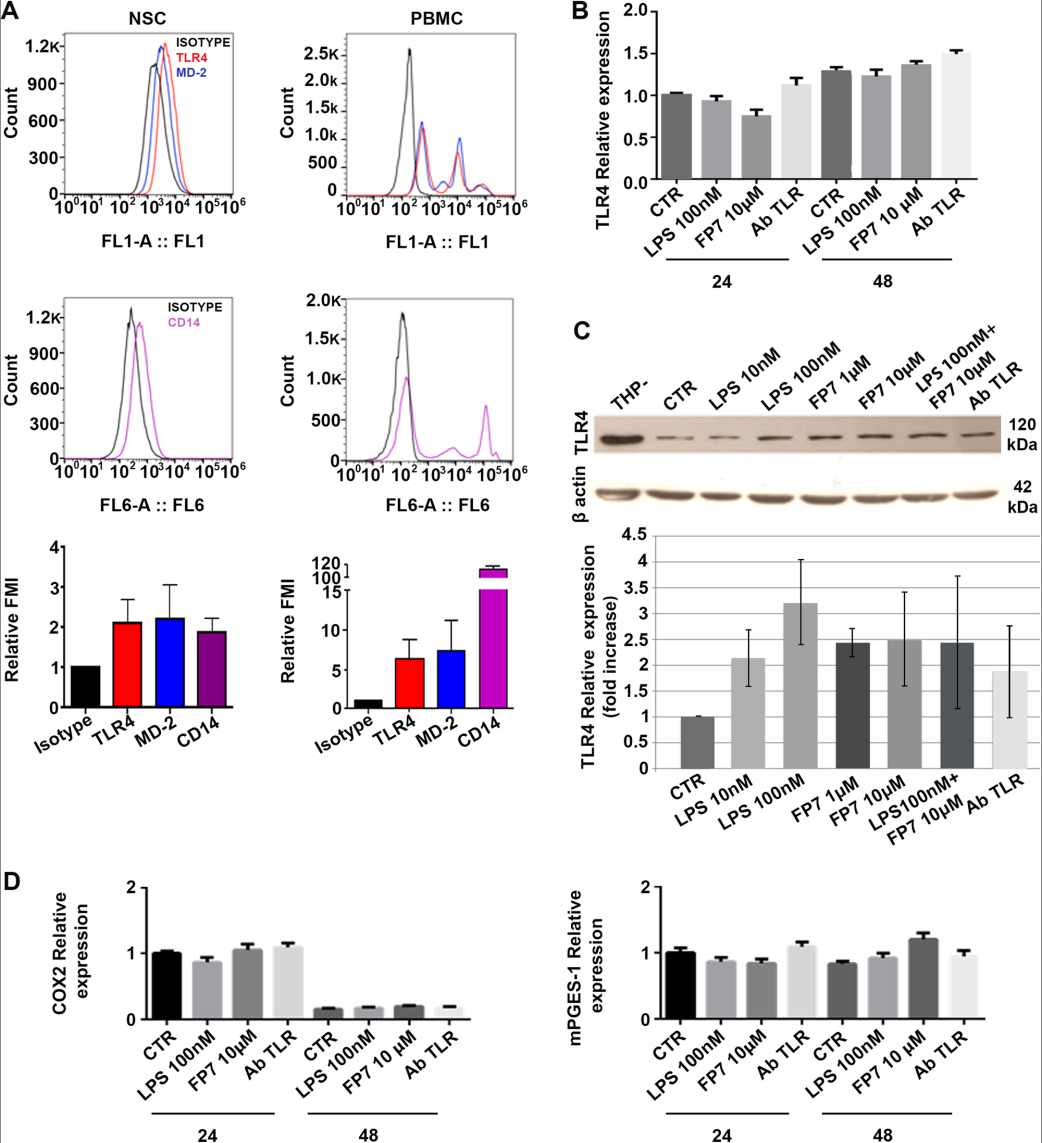
Expression of TLR4 in hNSC **a** Flow cytometric analysis of TLR4, MD-2 (top hystograms), and CD14 (bottom hystograms) surface expression on neural stem cells (left) and monocytes (right); Relative MFI quantification (bar graphs) of TLR4, MD-2, and CD14 surface expression in neural stem cells (left) and monocytes (right). Data are representative of two independent experiments. **b** Real-time PCR of *TLR4* gene in hNSC untreated or treated for 24 and 48 h with LPS 100 nM or FP7 10 μM. **c** Western blot analysis of TLR4 expression in THP-1 (positive control) cells and hNSC untreated or long-term treated with LPS 10 nM, LPS 100 nM, FP7 1 μM, or FP7 10 μM. **d** Real-time PCR of *COX2* and *mPGES-1* genes in hNSC untreated or treated for 24 and 48 h with LPS 100 nM or FP7 10 μM

Since both activation and inhibition of TLR4 signal pathway were shown to influence neurogenesis in the hippocampal brain^6,7^, we focused our attention on the controversial role of TLR4. By citofluorymetric analysis, the expression of TLR4, CD14, and MD-2 was detected in undifferentiated hNSC (dissociated neurospheres), but less than in peripheral blood mononuclear cells (PBMC) (positive control) (Fig. 1a). hNSC were then treated with LPS 10 and 100 nM and with the synthetic antagonist FP7 used at two concentrations, 1 and 10 μM^11,12^. In parallel, the cells were treated with a specific TLR4-blocking antibody (AbTLR4) as a control and co-treated with 100 nM LPS and 10 μM FP7 (maximal doses) to test the competition between the two molecules.

Real-time PCR analysis showed that the level of mRNA encoding TLR4 is easily detectable in hNSC neurospheres, with no variation after treating the cells for 24 and 48 h with saturating doses of LPS, or FP7 or AbTLR4 (Fig. 1b).

The prolonged administration (10–15 div) of LPS, FP7, and AbTLR4 throughout hNSCs amplification, showed some increase, though not significant, of the quantity of TLR4 protein in treated versus untreated cells, as illustrated by western blot analysis (Fig. 1c). In order to exclude that a CD14-dependent but TLR4-independent pathway might be involved in FP7-mediated effects, we evaluated by real-time PCR the expression of COX2 and mPGES-1, two downstream effectors of Nuclear Factor of Activated T-cells (NFAT) pathway^17^. No significant expression was detectable in untreated or treated hNSC neurospheres even at 24 or 48 h after dissociation, suggesting that the TLR4-indipendent NFAT pathway is not normally activated in hNSC (Fig. 1d).

**Fig. 2:**
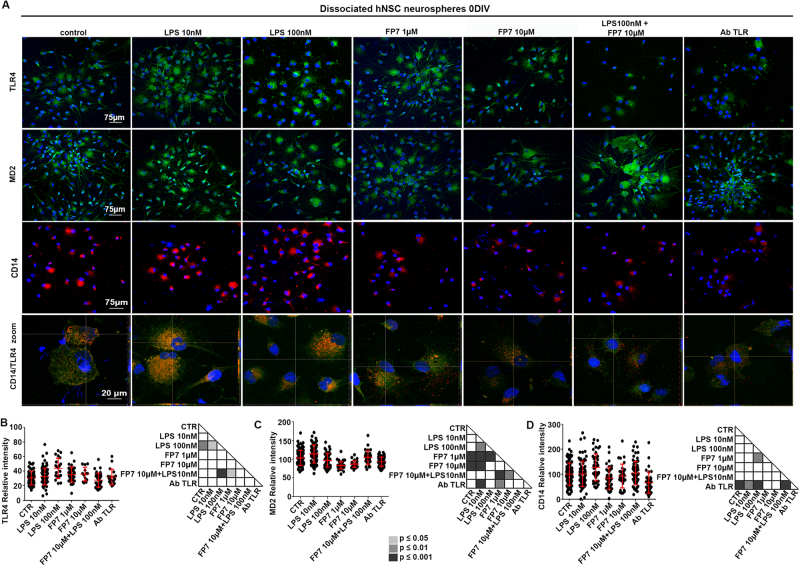
Modulation of TLR4 expression in hNSC by long-term treatment with LPS and FP7 **a** Confocal microscopy images of hNSC untreated or long-term treated as described in **b** and immunostained for TLR4 or MD-2 or CD14. Scale bar: 75 μm. **b–d** Scatter plots representing densitometric analysis of relative intensity of fluorescence of **b** TLR4 (*n* ≥ 17), **c** MD-2 (*n* ≥ 29), and (**d**) CD14 (*n* ≥ 31). Values are means ± SEM

Confocal microscopy analysis showed that TLR4 was heterogeneously expressed by the totality of the cells (Fig. 2a, b), with LPS 100 nM significantly enhancing TLR4 fluorescence intensity.

The levels of MD-2 was significantly reduced by treatment with the synthetic antagonist FP7 alone (Fig. 2c). A similar though more heterogeneous trend was obtained for CD14, with a significant reduction in hNSC treated with anti-TLR4 antibody (AbTLR4 Fig. 2a, d). Interestingly, LPS treatments seemed to enhance CD14 and TLR4 colocalization at subcellular level with respect to the other cells (Fig. 2a zoom).

These data suggest that TLR4 is endogenously expressed by hNSCs, and modulated with differentiation. Long-term treatment of hNSCs with either agonist or antagonist does not remarkably affect expression of TLR4, while may have different effects on the expression of the co-receptors MD-2 and CD14.

### TLR4 regulates hNSC proliferation

**Fig. 3:**
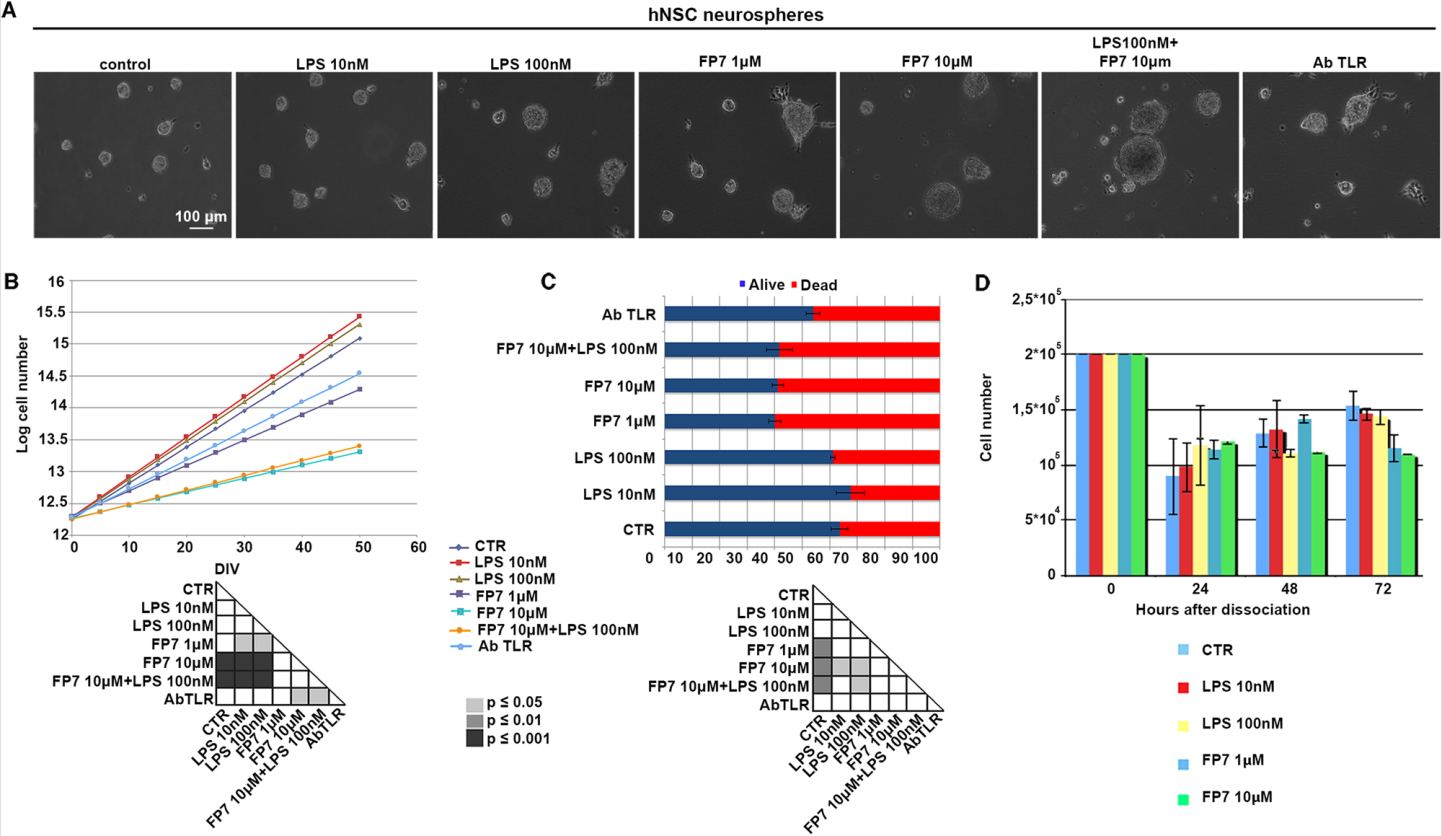
TLR4-mediated regulation of hNSC proliferation and self-renewal **a** Phase contrast images of hNSC neurospheres untreated or long-term treated with LPS 10 nM, LPS 100 nM, FP7 1 μM, or FP7 10 μM. Scale bar: 100 μm. **b** Growth curve analysis of hNSC untreated or treated as in **a**, **c** Quantitative analysis of the relative percentages of dead and alive hNSC after neurospheres’ dissociation and trypan blue staining. **d** Viability assay of hNSC after neurosphere dissociation (0 div) and 24, 48, and 72 h treatment with LPS 10 nM, LPS 100 nM, FP7 1 μM, or FP7 10 μM and relative statistical analysis. Values are means ± SEM

The role played by TLR4 in regulating NSC proliferation is still under debate^6,7,18^. To examine how TLR4 can affect hNSC proliferation, we treated hNSC cells with LPS and FP7 at different doses and characterized hNSC self-renewal capacity. Interestingly, hNSC (*n* = 2 lines) displayed a slight increase of the proliferation rate in the presence of LPS 10/100 nM, that was not significant (Fig. 3a, b). Conversely, a decrease of proliferation was observed in response to antagonist FP7 (1 μM) that became further significant at higher concentration (10 μM), indicating that TLR4 inhibition impairs proliferation of hNSC in a dose-dependent fashion. Interestingly, AbTLR4-treated cells displayed an effect similar to FP7 1 μM. FP7 10 μM effect was only partially rescued by co-treatment with LPS 100 nM, suggesting that inhibition of TLR4 by FP7 is able to counteract activation by either LPS or a putative endogenous ligand. The modest increase of proliferation triggered by LPS treatment suggests that TLR4 is normally activated in hNSCs in vitro and that it regulates their self-renewal and/or survival capacity, while inhibition of TLR4 exerts opposite effects, as assessed by staining with trypan blue after dissociation (Fig. 3c). To note, these effects were recognizable only after long-term treatment of hNSC. Indeed, a viability assay performed at 24, 48, and 72 h from neurospheres dissociation and administration of LPS or FP7 showed no significant difference between untreated and treated cells (Fig. 3d).

**Fig. 4:**
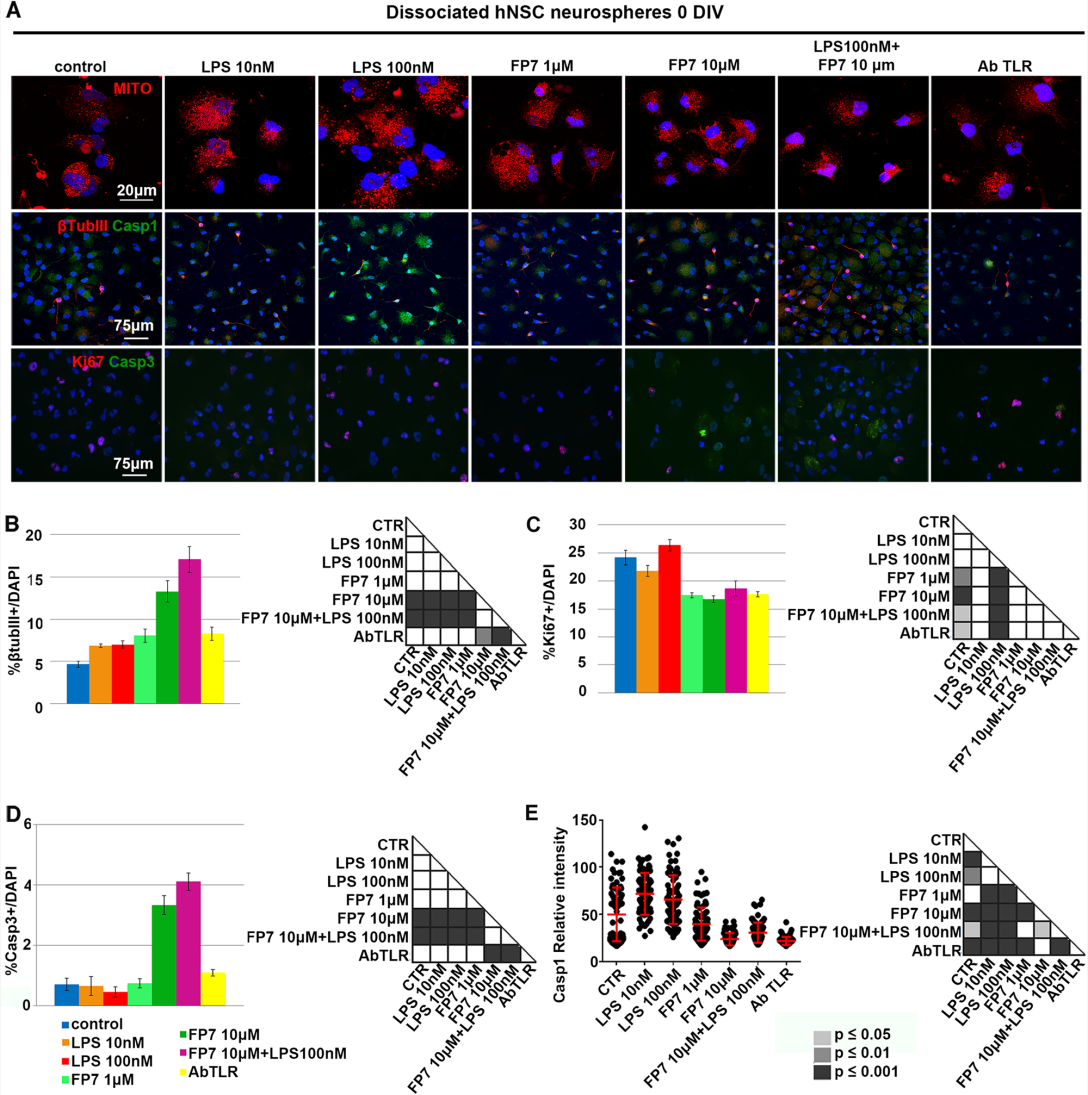
Mitochondrial pattern and differentiation markers in dissociated hNSC neurospheres after long-term TLR4 modulation **a** Mitotracker staining and immunofluorescence analysis of expression of βtubIII, Casp1, Ki67 and Casp3 markers of hNSC dissociated neurospheres after long-term treatment with LPS 10 nM, LPS 100 nM, FP7 1 μM, FP7 10 μM, LPS 100 nM+ FP7 10 μM and AbTLR. Scale bars: 20–75 μm. **b–d** charts showing the quantitative analysis of the percentage of βtubIII+ **b**, Ki67+ **c** and Casp3+ **d** cells over the total DAPI+ nuclei. Statistical significance is indicated. Values are means ± **e**SEM. Scatter distribution plot representing densitometric analysis of Casp1 relative fluorescence intensity (*n* ≥ 38 cells). Statistical analysis is indicated

To assess if long-term TLR4 inhibition could progressively shift the stem cells population to transiently amplifying progenitors, we dissociated long-term treated hNSC neurospheres and evaluated the expression of proliferation/survival markers and the precocious bias toward the neuronal lineage. In accordance with a lower proliferation profile, MitoTracker staining of mitochondria showed a reduced content of mitochondria in FP7-treated hNSC compared to untreated cells (Fig. 4a). Interestingly, a higher percentage of β-tubIII+ (early neuronal marker) progenitors was detected in FP7 10 μM-treated cells (Fig. 4a, b) together with a lower percentage of ki67+ cells (actively proliferating cells) (Fig. 4a, c) and a higher percentage of cells positive for Casp3 (an apoptotic marker) (Fig. 4a, d) compared to the other samples. On the contrary, LPS-treated hNSC displayed normal and abundant mitochondria (Fig. 4a), a number of ki67+ cells comparable to untreated hNSC and only sporadic apoptotic cells.

Since the inflammasome pathway has been emerging as a major player in cell innate immunity and metabolism^19^, we checked for the expression of Casp1 (Casp1), a marker of the inflammasome complex. Consistently with previous data^20^, LPS treatment induced a significant increase of Casp1 level, while FP7 and AbTLR4 led to a decrease of the protein (Fig. 4e and Fig. S4). These findings suggested that TLR4 agonist (LPS) and antagonist (FP7) may act through partially independent pathways. Agonist-mediated activation of TLR4 may support hNSC survival, while inhibition of TLR4 may play a role in the senescence process through accelerating transient proliferation of progenitors and differentiation.

To test how the effects observed in dissociated neurospheres were evolving in hNSC-deriving transiently amplifying progenitor cells (NPCs), we cultured dissociated hNSC neurospheres on adhesion for 3 div in FGF2 (Fig. S2). Compared to the treatments evaluated at 0 div (Fig. 4), LPS 100 nM significantly increased the fraction of Ki67+ proliferating cells (Fig. S2C), while FP7 decreased the percentage of β-tubIII+ cells (Fig. S2B) and increased the number of Casp3+ apoptotic cells (Fig. S2D), strengthening our hypothesis that TLR4 activation promotes neurogenesis while TLR4 inhibition progressively leads to cell death.

### TLR4 activation by LPS and inhibition by FP7 modulate the multipotency of hNSC

**Fig. 5:**
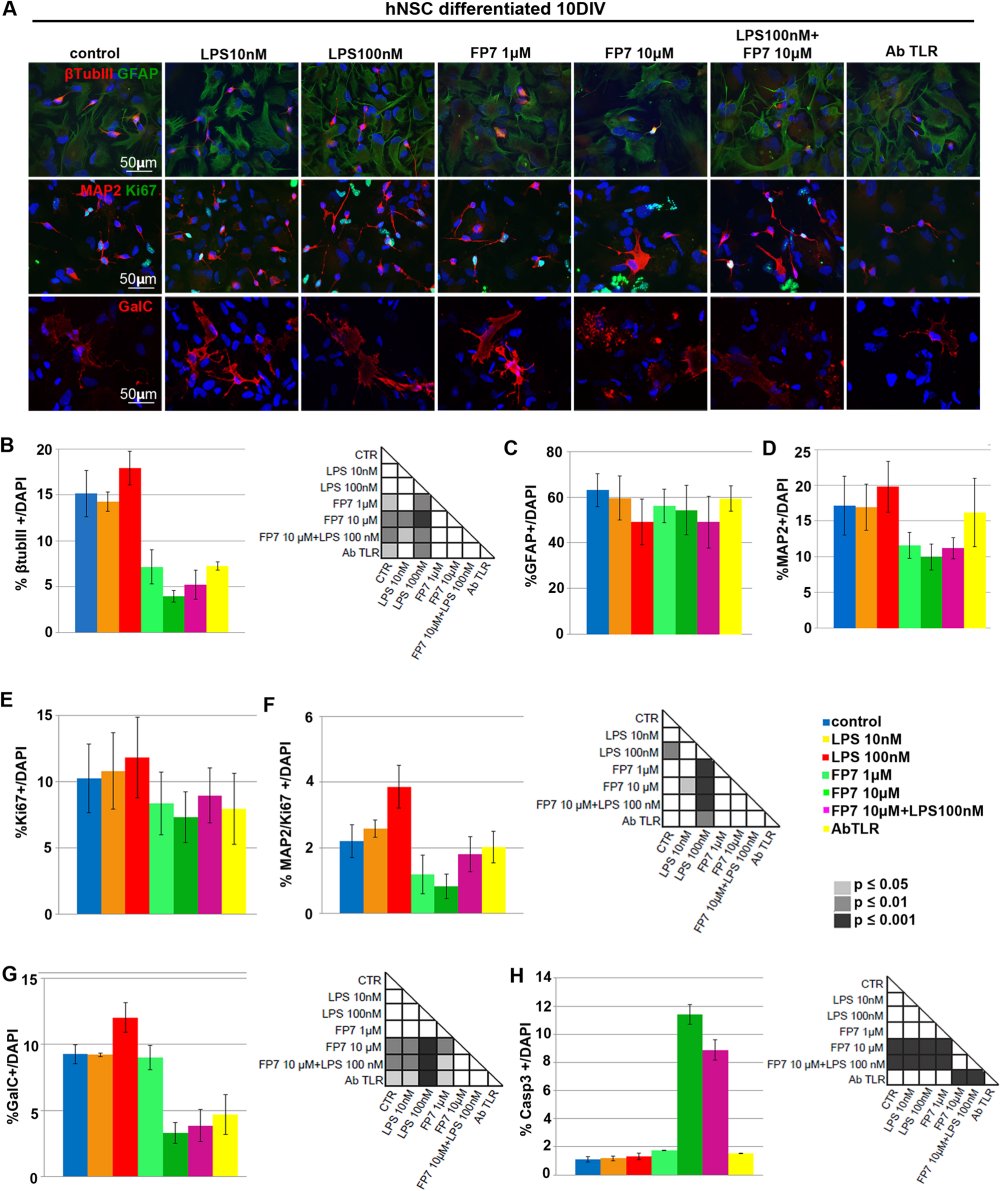
Effects of TLR4 modulation on early differentiation of hNSC **a** Immunofluorescence analysis of expression of βtubIII, GFAP, MAP2, Ki67, and GalC markers in hNSC differentiated at 10 div after treatment from 0 div with LPS 10 nM, LPS 100 nM, FP7 1 μM, FP7 10 μM, LPS 100 nM+ FP7 10 μM, and AbTLR block. Scale bars: 50 μm. **b–h** Charts showing the quantitative analysis of the percentage of βtubIII+ (**b**), GFAP+ (**c**), MAP2+ (**d**), Ki67+ (**e**), double-stained MAP2/KI67+ (**f**), GalC+ (**g**), and Casp3+ (**h**) cells over the total DAPI+ nuclei. Statistical significance is indicated for charts (**b**, **f**, **g**). Values are means ± SEM

**Fig. 6:**
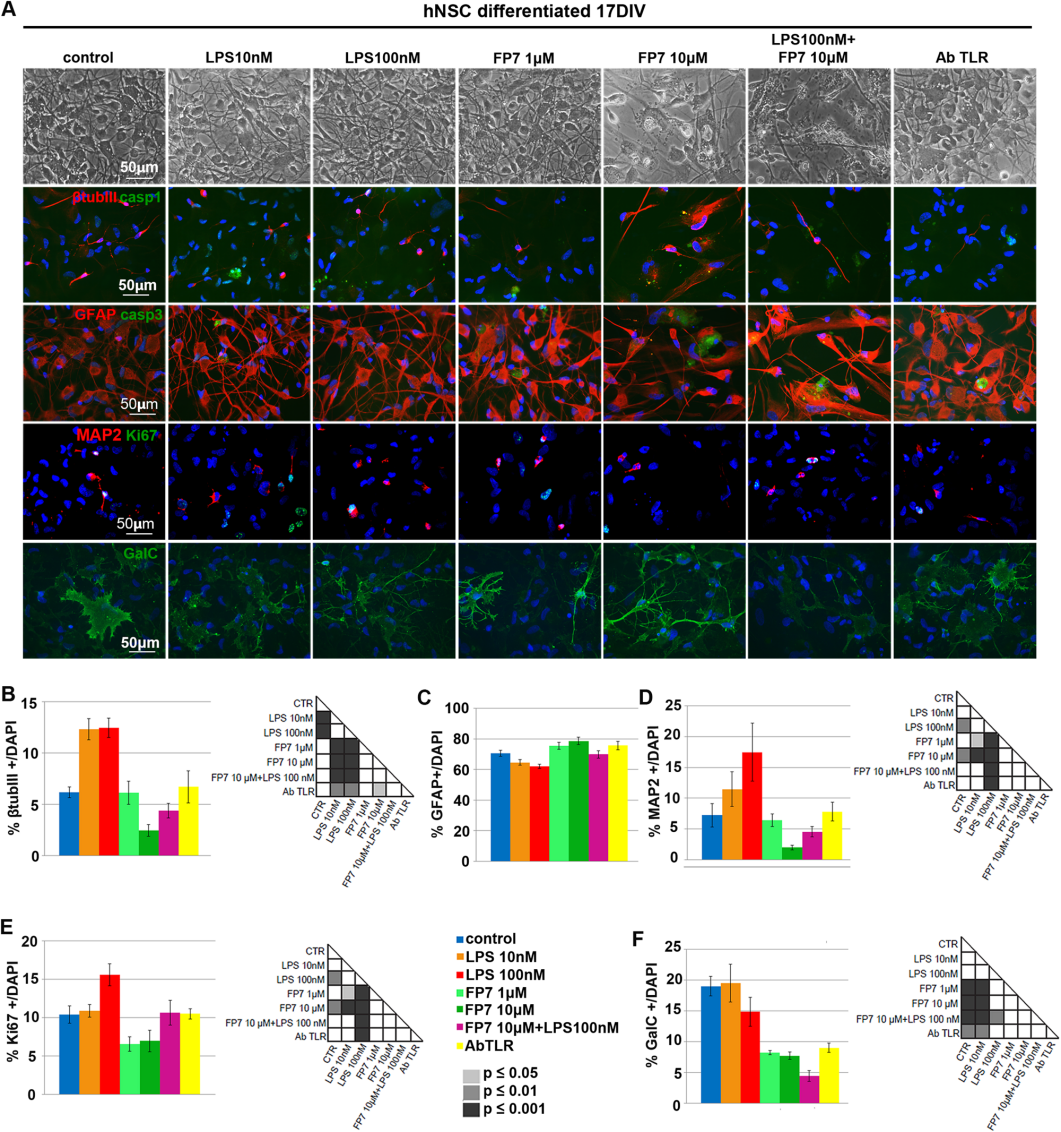
Effects of TLR4 modulation on late differentiation of hNSC **a** Phase contrast images and immunofluorescence analysis of expression of βtubIII+, Casp1, GFAP, Casp3, MAP2, Ki67, and GalC markers in hNSC differentiated at 17 div after treatment from 0 div with LPS 10 nM, LPS 100 nM, FP7 1 μM, FP7 10 μM, LPS 100 nM+ FP7 10 μM, and AbTLR block. To note, mature GalC+ oligodendrocytes are visible in FP7 10 μM-treated cells only sporadically. Scale bars: 50 μm. **b–g** Charts showing the quantitative analysis of the percentage of βtubIII+ (**b**), GFAP (**c**), MAP2 (**d**), Ki67+ (**e**), and GalC+ (**f**) cells over the total DAPI+ nuclei. Statistical significance is indicated for charts **b**, **d**–**f**. Values are means ± SEM

To evaluate the effects of TLR4 activation or inhibition on early differentiation of hNSCs, untreated hNSCs were differentiated in the treatments described above for 10 div and immuno-labeled for specific lineage markers: β-tubulin III (early marker) and MAP2 (late dendritic marker) for neurons, GalC for oligodendrocytes, and GFAP for astrocytes (Fig. 5a). LPS treatment did not affect either β-tubIII+ (Fig. 5b) and MAP2+ (Fig. 5d) neuronal or glial cells (Fig. 5c, g) compared to untreated. Differently, TLR4 inhibition by either FP7 (1 and 10 μM) or AbTLR4 blocking led to a decrease of β-tubIII+ cells. FP7 10 μM or AbTLR4 blocking also led to a decrease of GalC+ cells (Fig. 5a, b, g). Consistently, we found a higher percentage of cleaved Casp3+ in cells treated with FP7 10 μM compared to control, with no rescue by co-treatment with LPS 100 nM (Fig. 5h). The fractions of ki67+ cells had a trend that was complementary to that of Casp3+ cells (Fig. 5e), showing that proliferation delay during differentiation may be affected by the treatments. In particular, an increased percentage of residual proliferating (MAP2/Ki67+) neuronal progenitors was detectable in LPS 100 nM-treated cells. These data suggested that TLR4 activation promotes neuronal commitment and survival, while FP7-mediated TLR4 inhibition promotes transient proliferation followed by rapid differentiation and short-term survival of neuronal and oligodendroglial cells in a dose- and time-dependent fashion. To test this hypothesis, we differentiated hNSC up to 17 div (Fig. 6a–f). Fibrotic astrocytes and nearly absent recognizable neurons were evident in FP7 10 μM-treated cells (Fig. 6a). Consistently with previous analysis at 10 div, we could observe that both LPS 10 nM and 100 nM treatments were able to yield a higher percentage of β–tubIII+ neurons (Fig. 6b) and LPS 100 nM also a higher percentage of MAP2+ neurons (Fig. 6a, d), likely thanks to an increased survival ability and percentage of MAP2/Ki67+ neuronal progenitors (Fig. 5f). Conversely, FP7 10 μM induced a progressive decrease of neuronal (Fig. 6d) and oligodendroglial (Fig. 6f) cells and Ki67+ proliferating progenitors (Fig. 6e). FP7 1 μM and AbTLR induced a consistent reduction of the percentage of oligodendrocytes (Fig. 6f). Remarkably, a general downregulation of Casp1 level was evident in all differentiated cells (Fig. 6a and Fig. S4E) with respect to undifferentiated hNSC (Fig. 4a and Fig. S4C).

We then sought to determine if pre-treatment of hNSC during proliferation might enhance the effects observed in hNSC treated exclusively during differentiation. Hence, the hNSCs were long-term pretreated (10 div) with LPS or FP7 and then differentiated for 17 div (Fig. S3). LPS-pretreated hNSC generated a higher percentage of either β-tubIII+ (Fig. S3A, B) and MAP2+ cells (Fig. S3A, D) with respect to both untreated and non-pretreated cells (Fig. 6). The same beneficial effect was observed on the fraction of oligodendrocytes (Fig. S3G), with a rate of proliferation arrest comparable to control (Fig. S3E).

Conversely, FP7 pre-treatment enhanced neuronal demise (Fig. S3A, D) and proliferation arrest (Fig. S3A, E), causing a total depletion of MAP2/Ki67+ neuronal progenitors (Fig. S3E), and oligodendroglial degeneration. As a whole, these results confirm our driving hypothesis that TLR4 inhibition is detrimental to neurogenesis.

### TLR4 signaling in hNSC

Previous studies have shown that NF-κB is required for NSC initial differentiation^21^.

Since the NF-κB activation depends on upstream TLR4 signaling, we evaluated NFκB p65 expression by confocal microscopy following long-term hNSC challenge with LPS or FP7. NF-κB p65 was detectable in all the treatments (Fig. S4A) with no evident difference in the level of expression or translocation to the nuclei.

To assess the effect of TLR4 stimulation on NF-κB signaling cascade during differentiation, we evaluated the expression of the NFκB p65 and of its negative modulator IkBα in undifferentiated and early differentiated hNSC (10 div) by western blot analysis.

We observed that NF-κB p65 expression is not significantly different among treatments while IkBα is downregulated in response to FP7 in undifferentiated hNSC (Fig. 4d, e). On the other hand, no signal of either phospho-NF-κB p65 or phospho-IkBα (activated forms) could be detected, suggesting that NF-κB is not the major pathway activated downstream of TLR4 in our system. As expected, when we treated HEK293T cells with LPS 10 nM, we observed increasing phosphorylation of Nf-kB with complementary decreasing phosphorylation of IkBα from 10 to 20 min of LPS stimulation (Fig. S5).

Among the candidate mechanisms independent on NF-κB pathway, IRF3 and inflammasome are two notable TLR4 effectors. Only faint expression of IRF3 was detectable in neurospheres with no significant difference among treatments (Fig. S4A, C). On the contrary, whole mean fluorescence of Casp1 was altered with TLR4 stimulation or inhibition (Fig. 4E). When we examined by western blot analysis the expression of NLRP3, pro-Casp1 (p45) and active Casp1 (p20), that are key components of the inflammasome, FP7 10 μM led to a decrease of Casp1 p45 in neurospheres, while we detected no significant changes in NLRP3 expression or Casp1 p20 among the different treatments (Fig. S4F). Interestingly, an analogous trend could be observed during differentiation. In particular, while LPS enhances both the expression of Casp1 p45 and of the activated form Casp1 p20 compared to untreated cells, FP7 10 μM treatment leads to a decrease of p45 but sustains activation of the protein (p20 in Fig. S4G), suggesting that canonical inflammasome pathway may be differentially recruited upon consistent stimulation or inhibition of TLR4.

### TLR4-mediated alteration of metabolism in differentiating hNSC

**Fig. 7:**
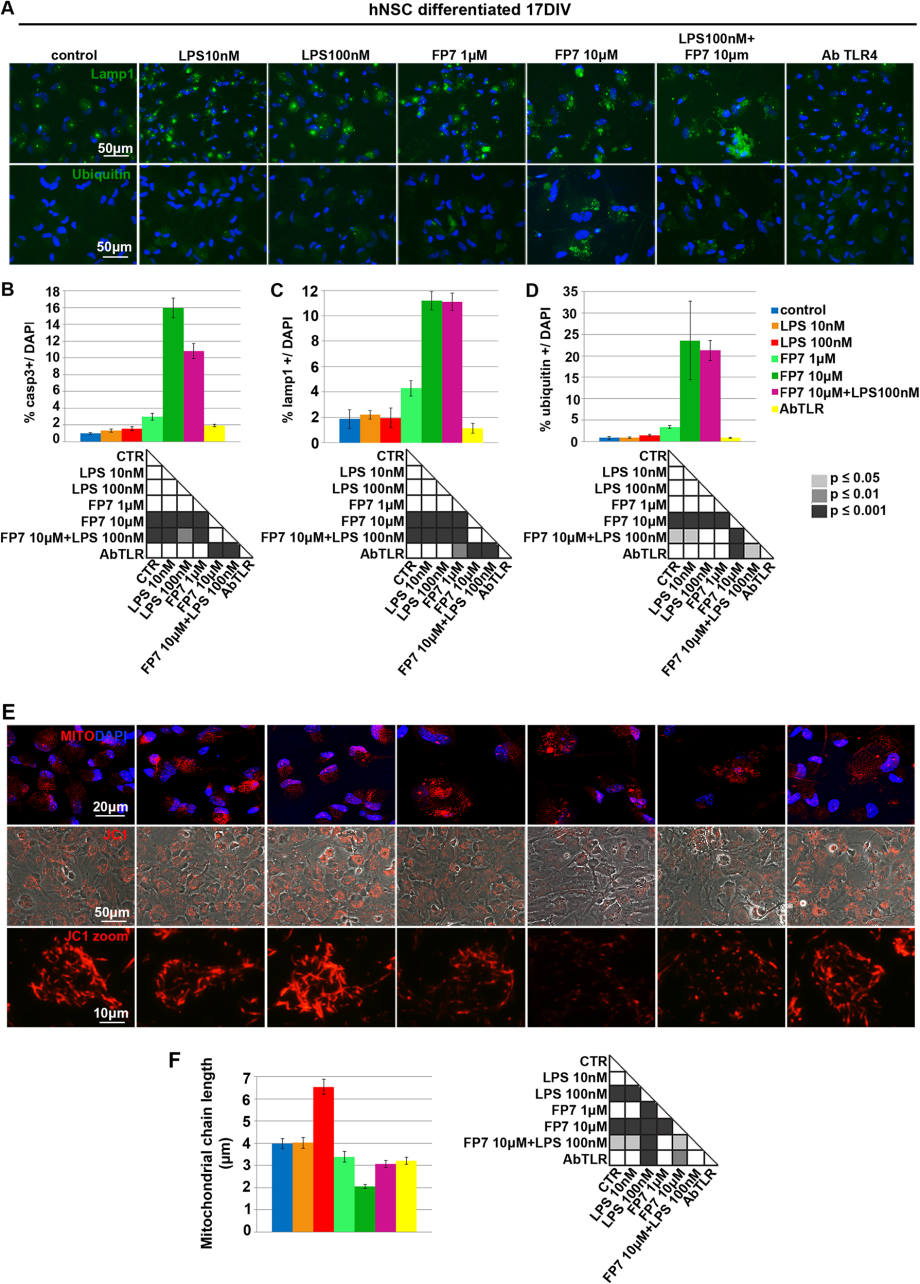
TLR4-mediated alteration of metabolic markers in differentiated hNSC **a** Immunofluorescence analysis of expression of Lamp1 and Ubiquitin metabolic markers in hNSC differentiated at 17 div after treatment from 0 div with LPS 10 nM, LPS 100 nM, FP7 1 μM, FP7 10 μM, LPS 100 nM+ FP7 10 μM and AbTLR block. Scale bars: 50 μm. **b–d** Charts showing quantitative analysis of the percentage of Casp3+ **b**, Lamp1+ **c** and Ubiquitin+ **d** cells over the total DAPI+ nuclei. **e**, **f** Fluorescence images showing staining of mitochondria in hNSC differentiated by Mitotracker and JC1 assay **e** and quantitative analysis of mitochondrial chain length **f** (*n* = 8-13). Scale bars: 10–50 μm. Statistical significance is indicated for charts **b**–**d**, **f**. Values are means ± SEM

The activation of TLR4 is known to be involved in the development of neuroinflammation *in vivo*, including processes like microgliosis and astrogliosis^22,23^. Morphological alterations of the cells are usually accompanied by a dysfunctional metabolism typically leading to aberrant degradation pathway and mitochondrial patterning^24^. Following the observation of changing morphology in cells treated with FP7 during differentiation (Fig. 6d, f), we investigated the metabolic pattern in hNSC differentiated at 17 div (as in Fig. 6) by immunofluorescence analysis with antibodies raised against Lamp1, a marker of the lysosomal/endosomal membrane (Fig. 7a, c) and ubiquitin (Fig. 7a, d). Consistently with an increase of Casp3+ cells (Fig. 7b), a significant increase of cells displaying lysosomal accumulation (Fig. 7a, c) and ubiquitin aggregation (Fig. 7a, d) was detectable in cultures treated with FP7 10 μM. Consistent, morphological analysis of mitochondrial pattern by Mitotracker and JC1 staining (Fig. 7e) illustrated a remarkable reduction of mitochondrial content and chain length in FP7-treated compared to untreated cells (Fig. 7e, f). These data suggest that TLR4 inhibition decreases the survival capacity with a progressive dysfunction of metabolism and a parallel increase of cell susceptibility and mortality.

### Expression of TLR4 is maintained by hNSC in vivo

**Fig. 8:**
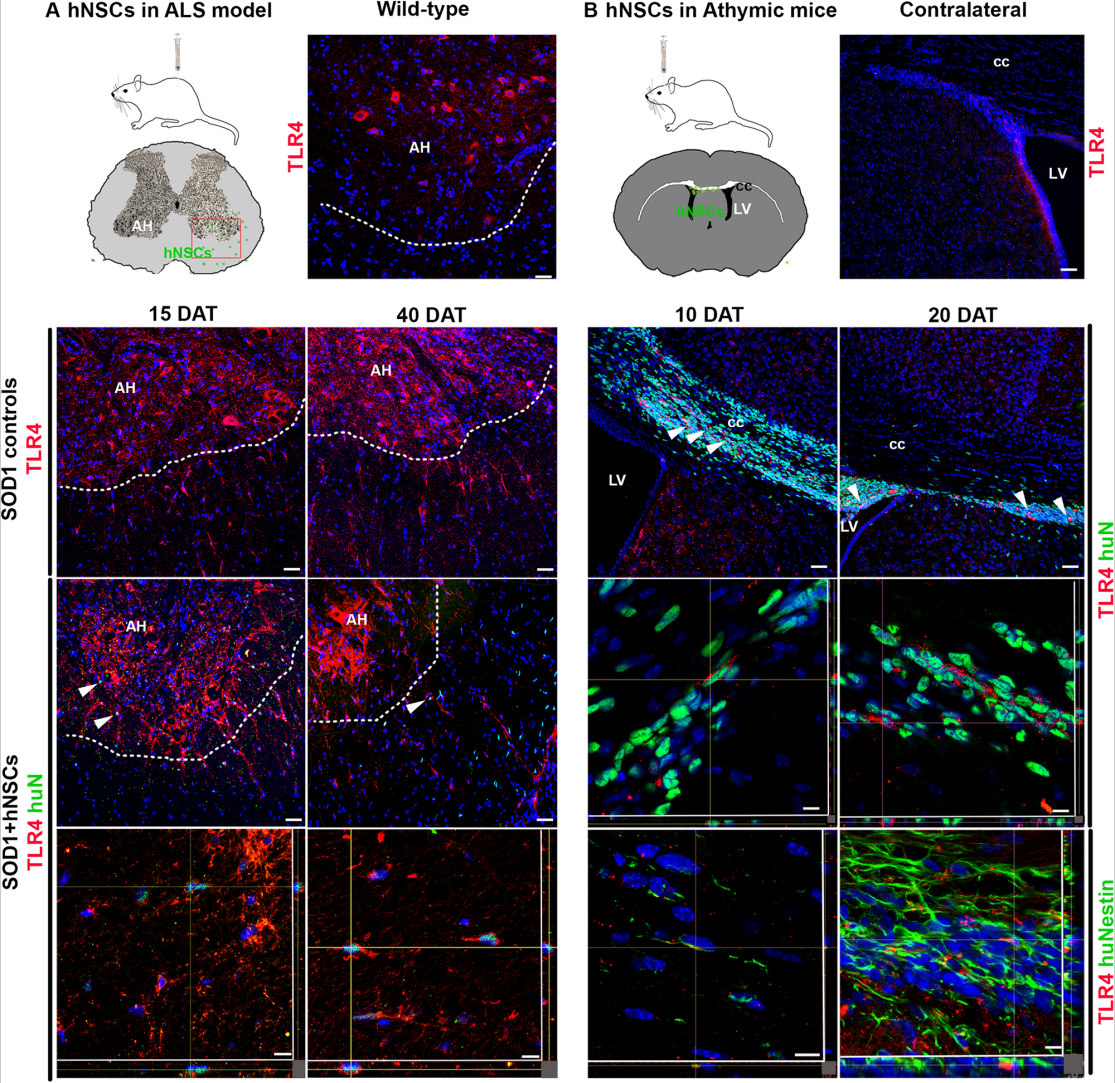
hNSC retain TLR4 expression *in vivo* **a** Left panel. Schematic of a coronal section of the spinal cord showing hNSCs (green dots) transplanted in anterior horns (AH) and migrating to surrounding regions. Expression of TLR4 (red) in L3–L4 tract of wild-type rats (upper panel, right), SOD1 not-transplanted controls (mid panel) and transplanted SOD1 rats (lower panel) at 15 and 40 DAT. Colocalization of TLR4 (red) with human-specific antigen (huN, green) or huNestin in transplanted hNSC is shown in the *z*-stacks (arrows, in lower panel). Scale bars: Upper, mid and lower panel: 40 μm, *z*-stacks: 10 μm. **b** Right panel. Schematic of a brain coronal section showing hNSCs (green dots) injected in the lateral ventricle (LV) and migrating into the corpus callosum (CC). Expression of TLR4 (red) in the mouse brain upper panel (right), and in hNSCs (arrows) at 10 (mid panel, left) and 20 (mid panel right) DAT. Colocalization of TLR4 with huN or huNestin is shown in the z-stacks (lower panels). Scale bars: Upper, mid and lower panel: 40 μm, z-stacks: 10 μm

In a translational view, it is of utmost importance to evaluate the modulation of TLR4 expression in hNSC *in vivo*, after transplantation into the CNS of animal models of neuroinflammatory diseases. We tested TLR4 expression in hNSC transplanted into the spinal cord of ALS (SOD1 G93A) rats at the early symptomatic stage. HBSS injected and wild-type animals were used as controls. The animals were followed up to 15 and 40 days after transplantation (15, 40 DAT) (late-symptomatic stage) and analyzed by co-immunostaining with anti-human nuclei (huN) and anti-TLR4 antibodies (Fig. 8). An overall increase of TLR4 expression was evident in SOD animals compared to wild type^16^. Confocal microscopy showed that hNSC were integrating and still expressing TLR4 at 40 DAT, suggesting that the maintenance of TLR4 expression may support hNSC survival after transplantation. To exclude that TLR4 expression in hNSC was induced by the neuroinflammatory environment, we examined expression of TLR4 in hNSC injected unilaterally in the brain of immunodeficient SCID mice. At 10 and 20 DAT, a discrete number of hNSC was expressing TLR4 together with nestin and mostly integrated in the SVZ (Fig. 8b, white arrows). These findings indicate that hNSC express TLR4 per se independently on the pathological environment and need TLR4 signaling for integration *in vivo*.

## Discussion

Here we investigated for the first time the effects of TLR4 modulation on *bona fide* hNSCs produced according to GMP guidelines and used in a clinical trial^15^.

hNSC transplantation currently represents a feasible strategy for the therapy of many neurodegenerative diseases or acute CNS injuries such as spinal cord trauma or ischemia, but a main roadblock is represented by the immunogenic potential of transplanted cells. CNS is considered an immune-privileged tissue, but the evident innate immune activity exerted by neurons and astrocytes have enlightened the neural cells as active players in the development of neuroinflammation together with non-neural components such as microglia and endothelial cells. We first showed that TLR4 and TLR2 are equally expressed in undifferentiated hNSC and their expression decreases with differentiation^25^. Hence, we focused our attention on the role of TLR4 in regulating the intrinsic properties of hNSC irrespectively of the non-neural components. To this aim, we evaluated the effects of TLR4 modulation in hNSCs *in vitro*. In particular, we treated hNSC by repeated stimulation with LPS and inhibition with a new, synthetic TLR4 antagonist (FP7) mimicking the chemical structure of lipid X, a natural monosaccharidic antagonist of TLR4. To the best of our knowledge, this is the first study providing evidence of expression of TLR4 and co-receptors MD-2 and CD14 on hNSC membranes under basal conditions. We showed that, though dynamic and heterogeneous, TLR4 expression is basically unaffected by either agonist (LPS) or antagonist (FP7), suggesting that similar to a variety of DAMPS ^26,27^, an endogenous ligand already induces expression of TLR4 possibly through a ligand-mediated positive feedback^28.^, FP7 inhibits TLR4 trafficking, thus prolonging the permanence of TLR4 on the membrane^11^.

In our study, TLR4 inhibition by FP7 antagonist significantly decreased hNSC proliferation rate in a dose-dependent fashion, while stimulation by LPS slightly increased hNSC self-renewal, suggesting that TLR4 is endogenously activated in self-renewing hNSC and that LPS is not toxic to hNSC in vitro. The identification of the endogenous TLR4 agonist(s) in the future would be pivotal to support hNSC survival after transplantation in cell-mediated therapies and to tailor appropriate therapeutic strategies to promote neurogenesis.

We have also shown that stimulation of TLR4 supports neuronal and oligodendroglial differentiation and survival, while TLR4 inhibition leads to opposite consequences. All these effects on differentiation of hNSC were enhanced if the cells were pretreated with LPS or FP7 during proliferation, confirming that TLR4 modulation affects both proliferating and differentiating hNSC in a time-dependent fashion and that the effect of TLR4 per se on hNSC cultured *in vitro* is not deleterious if isolated from other non-neural components such as microglia and macrophagic infiltrates^7^. We could not observe detectable activation of NF-kB or of its modulator IkBα, indicating that a NFκB-independent pathway may also be involved and that both long-term agonist and antagonist TLR4 modulations may drive hNSC to immunotolerant-like condition.

Interestingly, the concurrent modest increase of Casp1 levels in LPS-treated NSC is consistent with a basal activation of TLR4 and of the inflammasome Casp1/NLRP3 complex that does not impair survival and differentiation but may eventually precondition NSC sensitivity or regulate autophagy under physiological conditions^19–29^. Of note, this effect can be hindered by FP7 antagonist that activates the apoptotic pathway. A very recent study has shown that modulation of TLR4 expression is involved in the innate immune suppression of glioblastoma cancer stem cells (GBM CSCs) self-renewal such as in the responsiveness of non-CSCs to ligands^18^. Accordingly, we found that TLR4 is expressed in hNSC also *in vivo*, after transplantation and successful integration into the spinal cord of SOD1 rats. Importantly, similar results were obtained after injection of hNSC into the brain of uninjured immunocompromised mice, with co-expression of TLR4 with the early marker nestin (Fig. 8b) indicating that TLR4 expression in hNSC is not consequent to the pathological environment. Despite beyond the aim of the present study, future studies will be needed to assess the functional role of TLR4 in regulating the survival and engraftment capacity of hNSC after transplantation.

In conclusion, our findings strengthen the hypothesis that TLR4 plays a role in intrinsic proliferation and cell fate decision of hNSCs, independently of non-neural components present *in vivo*, and possibly on regenerative processes occurring under physiological and pathological conditions. These evidences open the way to possible therapeutic approaches for diseases hallmarked by progressive neuronal demise and reduced adult neurogenesis, representing an ever-increasing burden on health and social systems and services, with main reference to aging-related disorders.

## Materials and methods

### Cell culture and proliferation

hNSC (*n* = 2 lines) were cultured by neurosphere assay as described in De Filippis et al.^30^ with the addition of: LPS 10 nM or LPS 100 nM or FP7 1 μM and FP7 10 μM and AbTLR (0.5 μg/ml). Growth curve analysis and viability assay were performed as in De Filippis et al. Evaluation of viability over passaging was performed by trypan blue staining of dissociated neurospheres and counting of viable (bright) or dead (blue) cells over total cells. For each condition, the growth curves were performed in duplicate and generated comparable results.

### Differentiation of hNSC and immunocytochemistry

hNSCs were differentiated as described in ref.^30^ under different treatment conditions. Cells were fixed in 4% paraformaldehyde at 0, 3, 10, or 17 days along differentiation and processed for western blot (see below) or immunocitochemistry^30^ with primary (Supplementary Table S1) and secondary antibodies (Supplementary Table S2). Mitotracker and JC1 mitochondrial assay were performed as described in ref.^31^. Microphotographs were taken using a Zeiss Axiovert 200 direct epifluorescence microscope or with confocal microscope NIKON ECLIPSE T*i*.

### Reagents

*E. coli* LPS (O55:B5) was purchased from Sigma-Aldrich (#L2880). Molecule FP7 was synthesized in F. Peri labs. Anti-Human TLR4 mAb blocking (3C3) was a gift from Frank Neumann.

### Flow cytometric analyses

PBMCs freshly isolated from buffy coats of healthy donors by ficoll gradient and NSCs were resuspended in PBS with 5% FBS and 1:100 Fc block (30 min at room temperature (RT). Cells were then stained in PBS 5% FBS with anti-TLR4, anti-MD-2 (Supplementary Table S1) and anti-CD14 Alexa Fluor 647 (Supplementary Table S2) (30 min RT), washed twice with PBS and incubated with anti-rabbit Alexa Fluor 488 1:250 (Table S2) in PBS (30 min RT). Samples were washed twice incubated 1 min with 5 μg/ml of DAPI and immediately analyzed with Gallios flow cytometer. Analyses were performed with Flowjo X.

### Western blot analysis

Immunoblots were performed as described in De Filippis et al.^30^. Proteins were revealed by chemiluminescence (ECL, Amersham Biosciences AB) and detected on an X-ray film (Amersham Biosciences AB). Antibodies listed in Supplementary Table S1. Image processing and densitometric quantification were performed with using Image J.

### RNA extraction, cDNA synthesis, and qPCR analysis

Total RNA was extracted from 400,000 cells grown as described above using RNeasy mini kit (Qiagen). gDNA was removed on-column digestion, using RNase free DNase set (Qiagen). Reverse transcription was performed with 1 μg of total RNA using High-Capacity cDNA Reverse Transcription kit (Applied Biosystems, Thermo Fisher Scientific); we analyzed 90 ng per sample in triplicate. We performed qRT-PCR using TaqMan Gene Expression assay (Applied Biosystems, Thermo Fisher Scientific): *TLR4* (Hs00152939_m1), and *RPS13* (Hs01011487_g1), mPGES-1 (*PTGES*—Hs01115610), COX2 (*PTGS2*—Hs00153133), and *RPS13* (Hs01011487).

### Animal studies

hNSCs were injected into the spinal cord of SOD1 G93A transgenic male rats (*n* = 3) (SOD1 rats, Taconic, USA) and into the lateral ventricle of immunodeficient Athymic nude Foxn1/nu (*n* = 3). All animal care and experimental procedures were conducted according to the current *National and International Animal Ethics Guidelines* and approved by the Italian Ministry of Health.

hNSCs were bilaterally injected in the anterior horns of the L3–L4 segments of the spinal cord (4 sites, each 100,000 hNSCs/1 μl HBSS) in SOD1 rats at the early stage of the disease (∼post-natal day 110, p110)^32^. Animals were killed 15 and 40 days after transplant (DAT), corresponding, respectively, to a mid- and late-symptomatic phase of the disease. Wild-type (non-transgenic littermates), HBSS-treated SOD1 rats and disease stage-matched SOD1 control rats were also evaluated.

hNSCs (300,000) have been injected into one lateral ventricle of mice and animals killed at 10 and 20 DAT. Immunohistochemistry was performed on cryopreserved coronal spinal cord or brain sections fixed with PFA 4%. TLR4 expression in hNSCs was assessed through confocal colocalization of TLR4 (H-80) with the human-specific nuclear antigen, human Nuclei.

### Statistical analysis

Growth curves were analyzed with longitudinal linear model to assess trend over time. Time, treatment, and interaction term time-by-condition were included into the model. A *p* value <0.05 was considered statistically significant. All analyses were performed using SAS (SAS Institute, Cary, NC, USA).

Western blot data were analyzed using parametric Student’s *t* test and the statistical significance was designated as two-tailed *p* value <0.05.

For immunocytochemistry, data are reported as percentages of labeled cells over the total nuclei ± the standard error of the mean (SEM). An average of 3 × 10^3^ total cells (identified by DAPI nuclear staining) was counted randomly from two coverslips per condition in each experiment. Each value represents the average of three independent experiments. Data were analyzed by by Student’s *t* test and one-way ANOVA (Bonferroni test) and reported as mean ± SEM and are considered not statistically significant unless indicated in the figures (*p ≤ 0.05, **p ≤ 0.01, ***p ≤ 0.001).

Statistical analysis of densitometric studies from immunofluorescence images have been performed using GraphPad Prism (GraphPad Software, Inc., La Jolla, CA, USA). Most of populations have been demonstrated to have a non-Gaussian distribution, we therefore used the Dunn’s multiple comparison non-parametric test to perform the ANOVA.

## Electronic supplementary material

Supplementary Figure Legends

Supplementary Figure 1

Supplementary Figure 2

Supplementary Figure 3

Supplementary Figures 4

Supplementary Figures 5

Supplementary Tables of antibodies
