# Supplementary material for: Toll-like receptor 4 modulation influences human neural stem cell proliferation and differentiation
Source: Cell Death Dis. 2018 Feb 15;9(3):280. doi: 10.1038/s41419-017-0139-8 (PMC5833460; doi:10.1038/s41419-017-0139-8)
Supplement: Supplementary file 1 — Supplementary Figure Legends [file 41419_2017_139_MOESM1_ESM.docx]

**SUPPLEMENTARY FIGURE LEGENDS**

**Figure S1**

**Down regulation of TLR4 and TLR2 expression in hNSC with differentiation.**

Immunofluorescence analysis of expression of TLR2 and TLR4 in undifferentiated (0 DIV) and differentiated (10 DIV) hNSC after long-term treatment with LPS 100nM. Both TLR2 and TLR4 expressions undergo downregulation with differentiation, but only TLR4 expression is enhanced by LPS treatment. Scale bars: 100 μm.

**Figure S2**

**Effects of TLR4 modulation on hNSC-derived NPC**

hNSC were plated on adhesion and cultured for 3 DIV in the only presence of FGF2, in order to obtain a population of cells enriched of transiently amplifying progenitors (hNSC-NPC). **A**) Immunofluorescence analysis of expression of βtubIII, Casp1, Ki67 and Casp3 markers in hNSC. Scale bar: 100 μm. **B-D**) charts showing the quantitative analysis of the percentage of βtubIII+ (B), Ki67+ (C) and Casp3+ (D) cells over the total DAPI+ nuclei. Statistical significance is indicated. Values are means ± S.E.M.

**Figure S3**

**Effects of TLR4 modulation by LPS or FP7 are enhanced by pre-treatment of hNSC during proliferation.**

hNSC neurospheres were treated with LPS 10nM, LPS100nM, FP7 1μM, FP7 10μM, LPS100nM+ FP7 10μM and AbTLR block for 10 div, then dissociated and differentiated for 17 DIV under continuous treatment. **A)** Phase contrast images and immunofluorescence analysis of expression of βtubIII+, GFAP, MAP2, Ki67 and GalC markers. To note, mature GalC+ oligodendrocytes are visible in FP7 10μM treated cells only sporadically. Scale bars: 50μm.

**B-G)** Charts showing the quantitative analysis of the percentage of βtubIII+ (B), GFAP (C), MAP2 (D), Ki67+ (E), double stained MAP2+/Ki67+ (F) and GalC+ (G) cells over the total DAPI+ nuclei. Statistical significance is indicated. Values are means ± S.E.M.

**Figure S4**

**TLR4 modulation of NFkB and inflammasome pathway in undifferentiated and differentiated hNSC**

**A-C)** Grayscale confocal microscopy analysis of NFkB and IRF3 expression in undifferentiated hNSC after long-term treatment with LPS 10nM, LPS100nM, FP7 1μM, FP7 10μM, LPS100nM+ FP7 10μM and AbTLR block (A). Relative densitometric analysis of whole mean fluorescence is shown (B-C). Scale bars: 75μm.

**D-G)** Western blot analysis of NFkB, IkBα, NLRP3 and Casp1 (p45 and p20) expression in undifferentiated (D, F) and differentiated (E, G) hNSC. Quantification of relative protein expression is normalized to GAPDH expression.

**Figure S5**

**Activation of NfkB and IkBα in Hek 293T following stimulation with LPS**

Hek293T cells were stimulated with LPS 10nM for 10 and 20 min. Western Blot analysis of phosphorylated (A) and total (B) NFkB and IkBα expression shows an increase phosphorylation/activation of NFkB with concomitant decrease of phosphorylated IkBα. Quantification of relative protein expression is normalized to GAPDH expression.
