# Supplementary figures and images for: Toll-like receptor 4 modulation influences human neural stem cell proliferation and differentiation
Source: Cell Death Dis. 2018 Feb 15;9(3):280. doi: 10.1038/s41419-017-0139-8 (PMC5833460; doi:10.1038/s41419-017-0139-8)

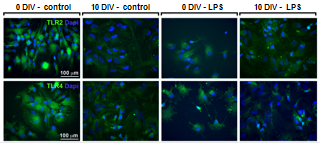

Supplement: Supplementary file 2 — Supplementary Figure 1 [file 41419_2017_139_MOESM2_ESM.tif]

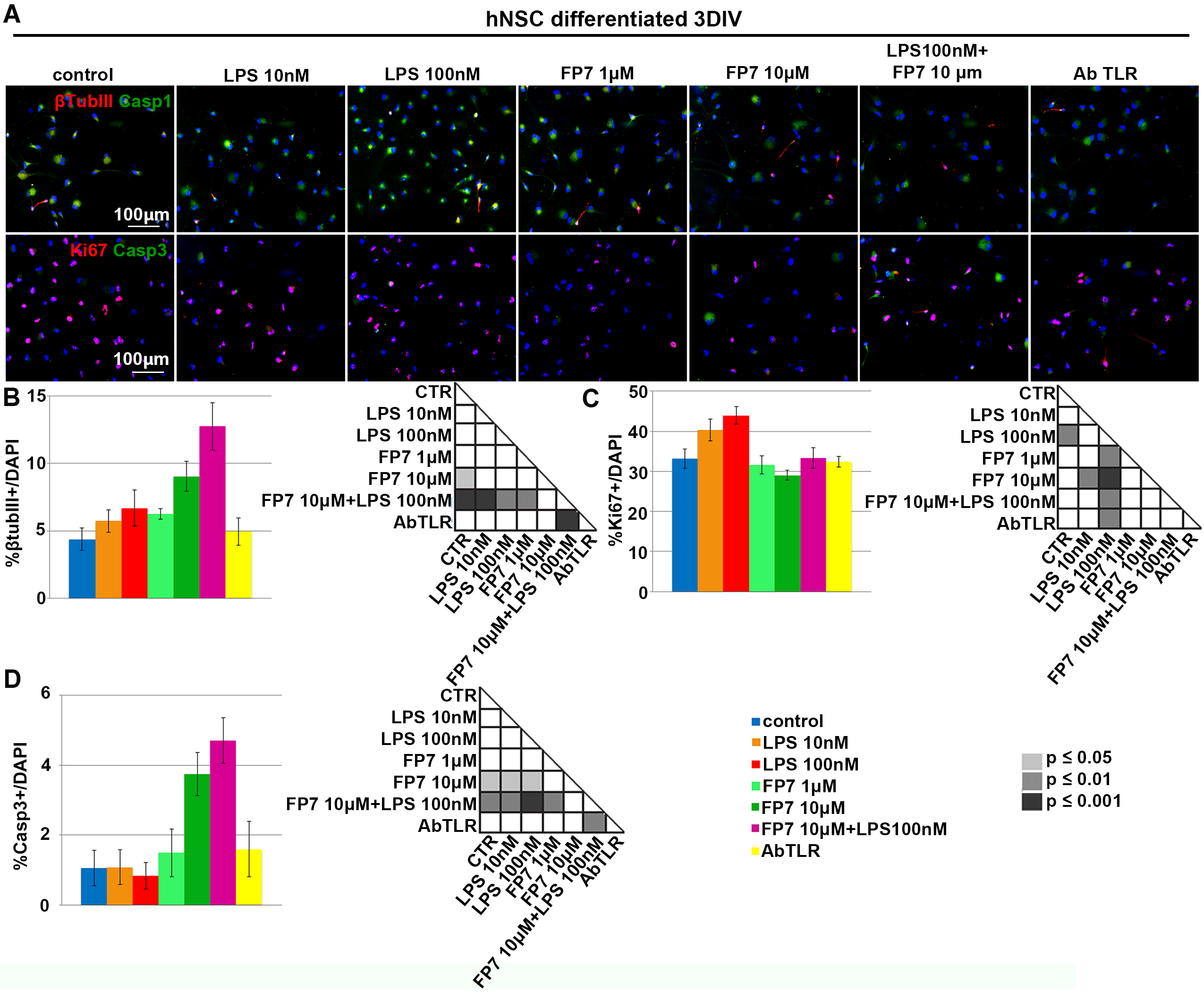

Supplement: Supplementary file 3 — Supplementary Figure 2 [file 41419_2017_139_MOESM3_ESM.tif]

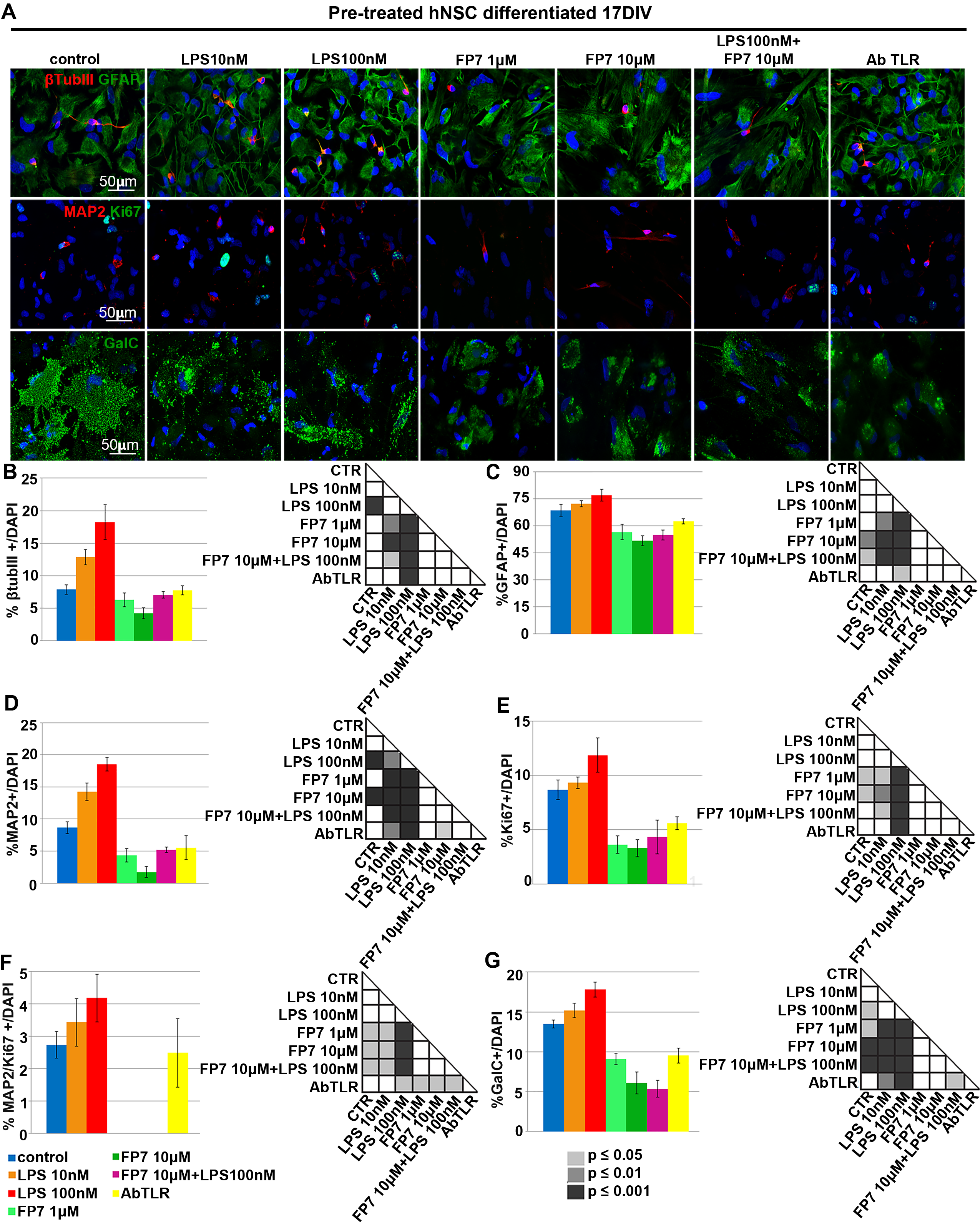

Supplement: Supplementary file 4 — Supplementary Figure 3 [file 41419_2017_139_MOESM4_ESM.tif]

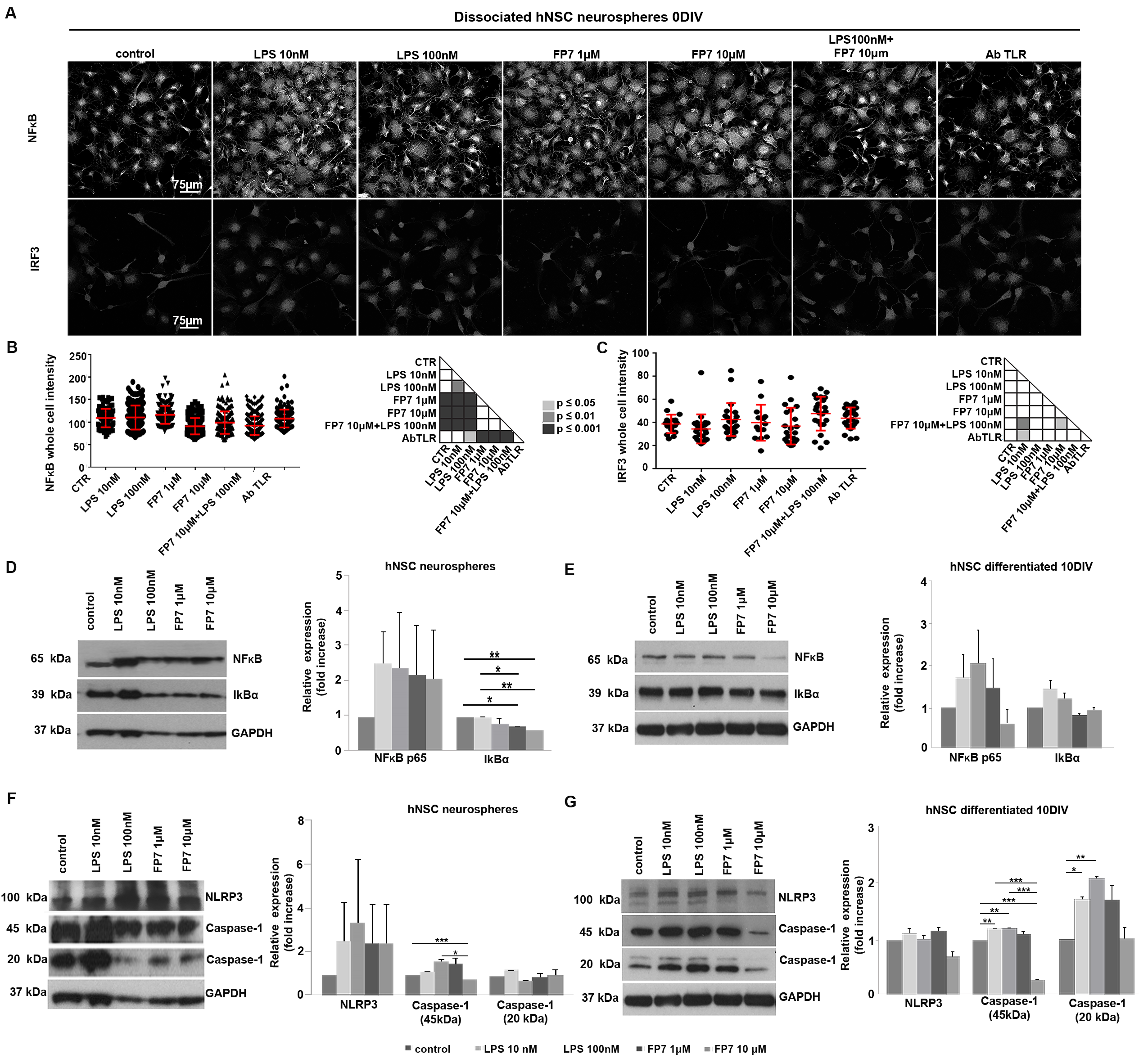

Supplement: Supplementary file 5 — Supplementary Figures 4 [file 41419_2017_139_MOESM5_ESM.tif]

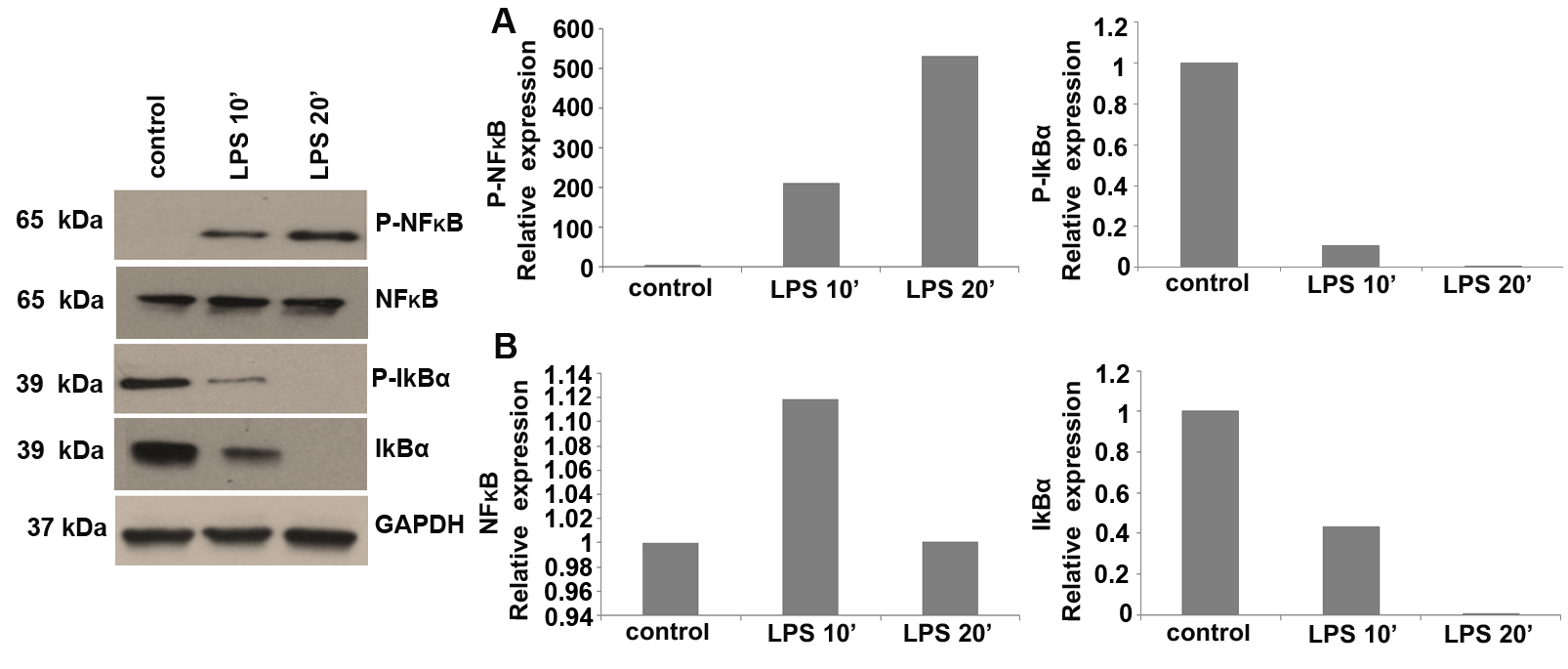

Supplement: Supplementary file 6 — Supplementary Figures 5 [file 41419_2017_139_MOESM6_ESM.tif]
