# Supplementary material for: Toll-like receptor 4 modulation influences human neural stem cell proliferation and differentiation
Source: Cell Death Dis. 2018 Feb 15;9(3):280. doi: 10.1038/s41419-017-0139-8 (PMC5833460; doi:10.1038/s41419-017-0139-8)
Supplement: Supplementary file 7 — Supplementary Tables of antibodies [file 41419_2017_139_MOESM7_ESM.docx]

Supplementary Table 1 Primary Antibodies

| Immuno-Fluorescence | | | |
| --- | --- | --- | --- |
| Antigen | **Supplier** | **Code** | **Concentration** |
| b-tubulin III (b -tubIII, mouse) | Covance | MMS-435P | 1:400 |
| Glial fibrillary acidic protein (GFAP, mouse) | Chemicon | MAB3402 | 1:500 |
| Glial fibrillary acidic protein (GFAP, rabbit) | Dako | Z0334 | 1:500 |
| Microtubular associated protein type 2 (MAP2, mouse) | Sigma | M9942 | 1:200 |
| Ki67 (rabbit) | Novus Biologicals | NB600-1252 | 1:500 |
| Galactocerebroside C (GalC, mouse) | Chemicon | MAB342 | 1:200 |
| Caspase-1 antibody (rabbit) | Cell Signaling | #2225 | 1:100 |
| Cleaved Caspase-3 antibody (rabbit) | Cell Signaling | #9661 | 1:500 |
| TLR4 (H-80) (rabbit) | Santa Cruz Biotechnology | sc-10741 | 1:150 |
| MD-2 (rabbit) | Santa Cruz | sc-20668 | 1:150 |
| Purified anti-human CD14 (mouse) | BioLegend | 301802/100μg | 1:400 |
| Human Nestin (hNestin, mouse) | R&D system | MAB1259 | 1:200 |
| Human Nuclei (hNuclei, mouse) | Millipore | MAB1281 | 1:100 |
| IRF-3 (rabbit) | Santa Cruz Biotechnology | sc-9082 | 1:100 |
| Western Blot | | | |
| TLR4 (H-80) (rabbit pAb) | Santa Cruz Biotechnology | sc-10741 | 1:200 |
| NF-κB p65 (D14E12, XP® rabbit mAb) | Cell Signaling | #8242 | 1:1 000 |
| IκB-α (L35A5, mouse mAb, Amino-terminal Antigen) | Cell Signaling | #4814 | 1:1 000 |
| Anti-NLRP3 /NALP3, mAb (Cryo-2) | AdipoGen | AG-20B-0014 | 1 μg/ml |
| NF-κB Pathway Sampler Kit | Cell Signaling | #9936 | 1:1 000 |
| Anti-Caspase-1 antibody [EPR4321] | Abcam | ab108362 | 1:1 000 |
| Caspase-1 antibody | Cell Signaling | #2225 | 1:1 000 |
| β-Actin (D6A8, rabbit mAb) | Cell Signaling Tech | #BK8457 | 1:1 000 |
| GAPDH (14C10, rabbit mAb) | Cell Signaling | #2118 | 1:1 000 |

Supplementary Table 2 Secondary Antibodies

| Immuno-Fluorescence |  |  |  |
| --- | --- | --- | --- |
| Secondary Antibody | **Supplier** | **Code** | **Concentration** |
| Cy2 | Jackson | 703-225-155 | 1:200 |
| Cy3 (against mouse or rabbit IgG) | Jackson | 711-165-152 | 1:800 |
| Alexa 546 (against rabbit IgG or mouse IgG1) | Molecular Probes | A11010 and A-21123 | 1:800 |
| Alexa 488 (against rabbit IgG or mouse IgG1) | Molecular Probes | A-11008 and A-21121 | 1:800 |
| Cy3 (against goat IgG) | Jackson | **705-165-147** | 1:1 000 |
| Western Blot |  |  |  |
| HRP (against mouse or rabbit IgG) | Cell Signaling | #7074 and #7076 | 1:2 000 |
